# Shorter mothers have shorter pregnancies

**DOI:** 10.1186/1687-9856-2015-S1-P109

**Published:** 2015-04-28

**Authors:** José Derraik, Tim Savage, Paul Hofman, Wayne Cutfield

**Affiliations:** 1 Liggins Institute, University of Auckland, New Zealand

## Objective

Studies have shown that shorter women are at a greater risk of preterm birth. In light of these previous findings, we aimed to assess whether maternal height was associated with gestational age in a cohort of children born at term.

## Methods

Subjects were a control cohort of 294 children of New Zealand European ethnicity, high socioeconomic status, naturally conceived, born appropriate-for-gestational-age of singleton pregnancies at 37–41 weeks of gestation. Gestational age was determined by ultrasound scans performed <20 weeks. Maternal and paternal heights were measured using a Harpenden stadiometer. Associations with maternal height were assessed using linear regression mixed models, accounting for infant's gender, birth order, maternal age, and paternal height, with family number added as a random factor.

## Results

Mothers were 167.5 (SD=6.2) cm tall (range 151.4–183.0 cm). Increasing maternal height was associated with longer gestation (p=0.002). Stratified analyses showed that the main effect appears to occur among shorter mothers (<165 cm tall), who experienced gestation that was ~0.6 weeks shorter than that of mothers 165–170 cm (p=0.001) and >170 cm (p=0.0002) tall (Figure 1). There was also a progressive increase in birth weight standard deviation scores with increasing maternal height (p<0.0001). Paternal height was not associated with study outcomes.

## Conclusions

This study shows that maternal height is positively associated with gestational length across the term window. An association between shorter mothers and preterm birth has been previously shown. We have extended this observation showing that maternal short stature is associated with shorter pregnancies within the term window. The breakdown of term pregnancies into early term (37 0/7 – 38 6/7 weeks of gestation), full term (39 0/7 – 40 6/7 weeks), and late term (41 0/7 – 41 6/7 weeks) has recently been proposed, in light of differences in neonatal morbidity and mortality as well as in neurocognitive outcomes later in life.

Maternal stature seems to be a factor determining offspring outcomes, with proposed underpinning mechanisms including socioeconomic factors, undernutrition in utero, and anatomical constraints on pelvis during birth. In our study, socioeconomic factors were not at play in light of the homogeneity of our cohort. Lastly, our findings also support the lack of a paternal height effect on length of gestation.

